# Oral Cavity Antibacterial Discovery Pipeline Driven by Advanced Analytical Techniques

**DOI:** 10.3390/molecules31162804

**Published:** 2026-08-12

**Authors:** Antonella Maria Aresta, Giada Stefania Signorile, Antonietta Clemente, Nicoletta De Vietro, Carlo Zambonin

**Affiliations:** 1 Department of Biosciences, Biotechnology and Environment, University of Bari, Via Edoardo Orabona, 70125 Bari, Italy; nicoletta.devietro@uniba.it (N.D.V.); carlo.zambonin@uniba.it (C.Z.); 2 Department of Clinical and Experimental Medicine, University of Foggia, via Napoli 121, 71122 Foggia, Italy; giadasignorile@gmail.com (G.S.S.); antoniettaclemente@gmail.com (A.C.)

**Keywords:** oral microbiome, bioactive metabolites, biomarker discovery, antibacterial discovery, LC-mass spectrometry, imaging mass spectrometry

## Abstract

The oral cavity represents an extremely complex and dynamic microbial ecosystem capable of rapidly adapting to antimicrobial stress. These characteristics make it a promising environment for the identification of novel bioactive molecules with therapeutic potential. At the same time, the increasing prevalence of resistant pathogens in dental and oral-maxillofacial infections highlights the limitations of current therapeutic strategies and the urgent need for new effective antibacterial agents. This review examines the central role of advanced analytical techniques, with particular emphasis on mass spectrometry, in the discovery and characterization of bioactive metabolites and biomarkers relevant to future antibacterial discovery and to the understanding of biological responses to pathogens or therapeutic interventions. It discusses how the integration of metabolomics approaches, imaging mass spectrometry, and bioinformatics platforms is transforming the antibacterial discovery process by accelerating the identification of active compounds and improving the understanding of microbial interactions within the oral cavity. Overall, this work provides an up-to-date overview of current knowledge regarding the oral microbiome as a source of bioactive molecules and biomarkers. It highlights how emerging analytical technologies are opening new perspectives for the development of innovative therapeutic strategies against resistant oral infections.

## 1. Introduction

Antimicrobial resistance (AMR) represents a growing global public health challenge, with a steady increase in morbidity, mortality, and economic burden associated with drug-resistant infections, as documented by global burden estimates [1], clinical and epidemiological reports [2], mechanistic reviews [3], and policy-level analyses [4]. Although AMR has traditionally been associated with nosocomial or systemic infections, increasing evidence indicates that the oral cavity plays a more central role than previously recognized. The oral microbiome is a complex and highly organized community in which microorganisms living in proximity establish synergistic and antagonistic interactions that determine health or disease [5]. Its high microbial density and continuous exposure to environmental and therapeutic selective pressures make the oral cavity a potential hot spot for the emergence and dissemination of antimicrobial resistance traits [6]. Because of these characteristics, the oral microbiome, and particularly the oral metaproteome, has been extensively investigated as a source of functional biomarkers for both oral and systemic diseases, as demonstrated by early metaproteomic characterizations [7], clinical proteomic surveys [8], large-scale functional profiling studies [9,10,11], and recent integrative multi-omics analyses [12]. Proteomic signatures reflect not only taxonomic shifts but also the functional state of the microbial community, providing insights that extend beyond those obtainable through metagenomics alone. However, despite the increasing genomic characterization of the oral resistome, a functional understanding of resistance determinants and their actual metabolic expression remains limited. Metagenomic analyses alone are insufficient to identify the antibacterial metabolites effectively produced within the oral biofilm.

Recent studies have further emphasized the remarkable microbial and metabolic diversity of oral biofilms, suggesting that this environment may also serve as a reservoir of bioactive molecules with ecological and therapeutic relevance [13]. Metagenomic investigations have revealed a rich and dynamic oral resistome characterized by a large repertoire of antimicrobial resistance genes (ARGs) and mobile genetic elements (MGEs), as well as by the ability of oral microorganisms to accumulate and exchange resistance determinants [14]. Differences in resistome composition among healthy subjects and patients affected by caries or periodontitis indicate that the oral microbial community is highly responsive to environmental and therapeutic pressures [15]. Addressing this gap requires not only high-resolution analytical approaches but also improved genetic tractability of host-associated bacteria. As highlighted by Waller et al. [16], the development of robust genetic tools is essential for the functional validation of resistance determinants and for elucidating the biosynthetic pathways underlying the production of bioactive metabolites. In this context, the need for an integrated framework capable of linking genetic potential to the actual production of bioactive metabolites becomes increasingly evident. Clinically, the growing detection of resistant pathogens in dental and oral-maxillofacial infections underscores the limitations of current therapeutic strategies and the urgent need for novel antibacterial agents [17]. The oral cavity therefore represents not only an underestimated reservoir of antimicrobial resistance, but also a promising source of novel antibacterial molecules.

In this scenario, mass spectrometry (MS)-based analytical techniques are emerging as transformative tools for the discovery and characterization of antibacterial metabolites within the oral ecosystem. Their high sensitivity and resolution enable the identification of bioactive compounds produced by both commensal and pathogenic microorganisms, the profiling of metabolic responses to antimicrobial stress, and the detection of rare or previously uncharacterized metabolites. MS-based approaches make it possible to directly link genetic potential to the actual production of bioactive metabolites, thereby overcoming some of the limitations of traditional genomic analyses. In addition, the integration of imaging mass spectrometry is reshaping antibacterial discovery in the oral cavity by providing new opportunities for the development of innovative therapeutic strategies against periodontal disease, peri-implant infections, and other biofilm-associated conditions.

This review provides a conceptual framework for understanding how MS-based approaches are reshaping antibacterial discovery within the oral ecosystem. Although the oral microbiome includes bacteria, fungi, archaea and viruses that interact within highly structured biofilm communities, the present review focuses primarily on bacterial communities. This choice reflects the current state of the field, where MS-based antibacterial discovery channels have been developed predominantly around bacterial metabolites, proteins and biofilm-associated mechanisms, while comparable applications to other microbial realms remain relatively unexplored [18]. In particular, fungal taxa such as *Candida* spp. represent an important component of the oral microbiome [19]; however, MS-based metabolomic and proteomic workflows, specifically adapted to oral fungi, remain limited [20]. For this reason, fungal communities are not extensively covered in this review, although their ecological and pathogenic relevance underscores the need for future studies.

First, we describe the evolution of antibacterial discovery pipelines and the current understanding of the oral resistome and its functional expression, highlighting the limitations of traditional discovery strategies and the technological advances driving this transition. We then examine the principal MS-based analytical platforms for antibacterial discovery, including metaproteomics, untargeted metabolomics, imaging mass spectrometry (IMS), and molecular networking, critically discussing their analytical capabilities, methodological limitations, and emerging applications in oral microbiology. Finally, we explore how the integration of these complementary technologies is enabling the identification of novel antibacterial metabolites and therapeutic targets, and discuss the remaining challenges for translating these discoveries into innovative strategies against biofilm-associated oral infections.

## 2. The Antibacterial Discovery Pipeline

The discovery of new antibacterial agents has become one of the most urgent priorities in biomedical research [21,22]. In this context, the antibacterial discovery pipeline can be defined as a structured decision-making framework that has progressively evolved in response to technological advances and conceptual shifts. Rather than following a strictly linear sequence of experimental steps, modern discovery increasingly relies on an iterative, modular, and data-driven strategy in which microbiology, analytical chemistry, pharmacology, computational biology, and multi-omics approaches operate in close synergy.

Traditionally, antibacterial discovery followed a sequential workflow, progressing from phenotypic screening to hit identification, lead optimization, and finally pharmacokinetic and toxicological evaluation. Although this strategy led to the discovery of many clinically successful antibiotics, it is no longer sufficient to address current therapeutic challenges. The rapid emergence of multidrug-resistant bacteria, together with the limited number of new antibacterial classes and the progressive exhaustion of easily druggable targets, has exposed the limitations of the classical discovery paradigm. Recent analyses have shown that the traditional pipeline often lacks effective integration between microbiology, medicinal chemistry, pharmacology, and translational biology, resulting in poor predictive performance and high attrition rates during preclinical and clinical development [23,24]. Consequently, despite substantial research efforts, the current antibacterial pipeline remains critically depleted of candidates with truly novel mechanisms of action capable of addressing the growing burden of antimicrobial resistance [25].

To overcome these limitations, a modular, iterative, and decision-driven discovery framework has progressively emerged. Waller et al. [16] proposed an emblematic model in which early target validation, mechanism-of-action elucidation, quantitative permeability assessment, and functional vulnerability analyses are incorporated from the earliest stages of development. This strategy promotes continuous feedback between chemistry, microbiology, pharmacology, and systems biology, thereby improving decision-making and reducing late-stage failures.

**Figure 1:**
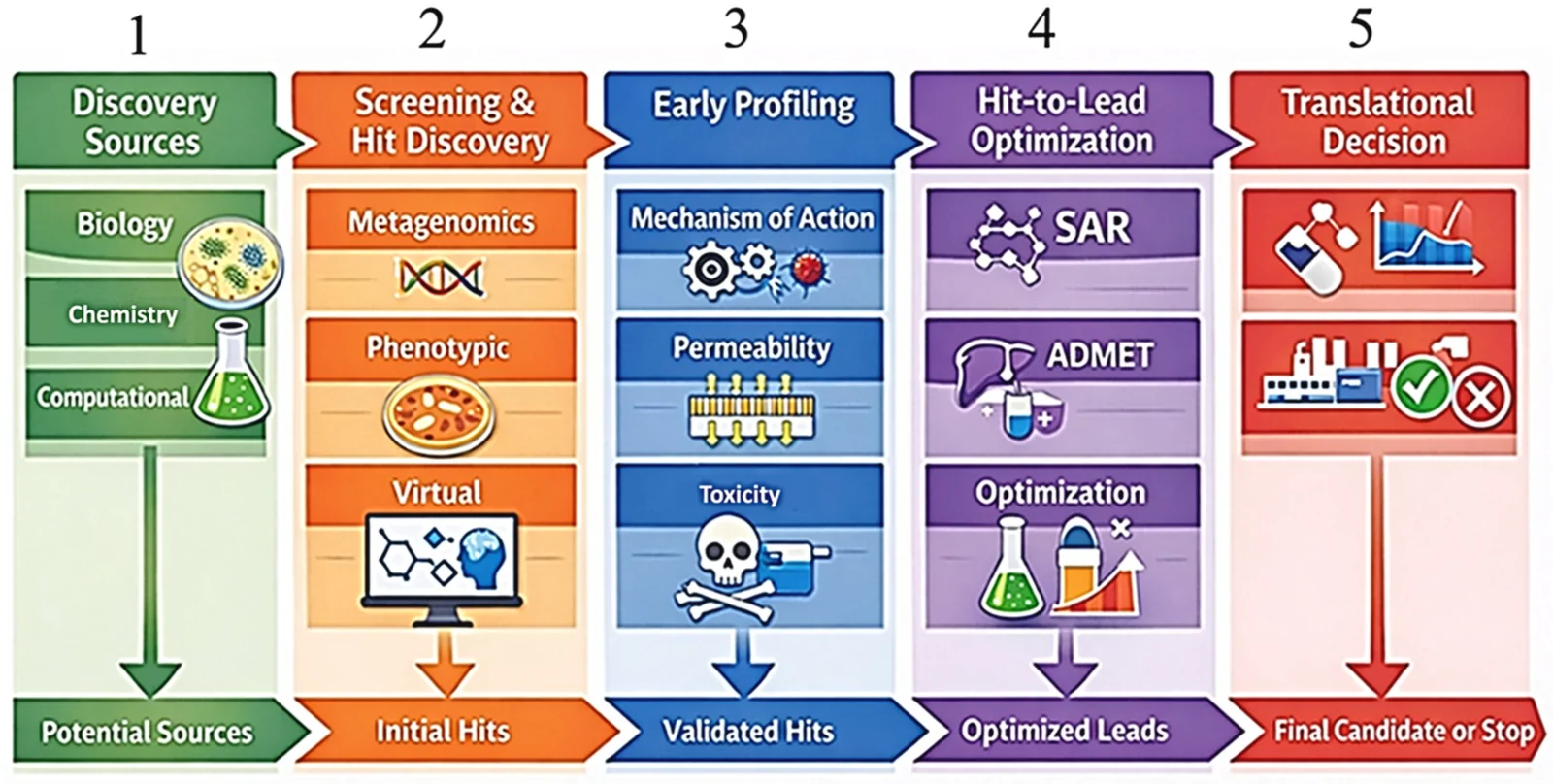
Schematic representation of the modular antibacterial discovery pipeline. The process includes five interconnected stages: (1) selection of discovery sources; (2) screening and hit discovery, generating the first biological signals and identifying initial hits; (3) early validation, integrating information on mechanism of action, permeability, and toxicity; (4) hit-to-lead optimization, in which hits are refined through structure–activity relationship (SAR) studies and ADMET evaluation (absorption, distribution, metabolism, excretion, and toxicity); and (5) translational decision-making, where scalability, chemical novelty, and potential clinical impact are assessed.

As summarized in Figure 1, this conceptual transition is based on five interconnected modules that continuously exchange biological and analytical information throughout the discovery process. Rather than relying exclusively on phenotypic activity, candidate prioritization is guided by multiple complementary layers of evidence, including target engagement, metabolic response, molecular profiling, and pharmacological properties. The integration of multi-omics technologies, advanced mass spectrometry, bioinformatics, and machine-learning approaches further enhances this framework by enabling the early identification of promising antibacterial candidates while reducing the progression of nonspecific or poorly translatable compounds. Moreover, quantitative assessments of permeability, pharmacokinetics/pharmacodynamics (PK/PD), and target vulnerability can now be incorporated during the pre-hit validation stage, substantially improving the predictive value of the discovery pipeline.

High-resolution analytical technologies, particularly liquid chromatography–mass spectrometry (LC-MS/MS), high-resolution mass spectrometry (HRMS), imaging MS approaches such as matrix-assisted laser desorption/ionization (MALDI) and desorption electrospray ionization (DESI), ion mobility–mass spectrometry (IM-MS), quantitative proteomics, and global metabolomics, now play a central role in this framework. These technologies enable the measurement of intracellular accumulation and biotransformation, the association of metabolic profiles with mechanisms of action, the identification of functionally vulnerable pathways, and early dereplication, thereby drastically reducing false positives. However, the robustness of the results strongly depends on the quality of both analytical and computational workflows. Preprocessing steps, including peak detection, alignment, normalization, and deconvolution, introduce substantial variability and represent a major challenge for reproducibility [26].

Wang et al. [27] addressed a major limitation in MS-based research, namely the lack of open and collaborative tools for sharing, searching, and curating raw instrumental data. As highlighted by several methodological reviews, the principal bottleneck no longer lies in instrumentation itself, but rather in the interpretation and management of the large amount of data generated. Existing databases often do not support searches directly based on raw MS spectra. To address this issue, the authors developed GNPS (Global Natural Products Social Molecular Networking), an online platform designed to enable MS data sharing, molecular networking, collaborative curation, and natural product discovery. Similarly, Rochat [28] emphasized that HRMS platforms such as Orbitrap and quadrupole time-of-flight (Q-TOF) instruments enable highly powerful omics analyses but require accurate and carefully curated interpretation. Perez de Souza et al. [26] further highlighted how the complexity of UHPLC-HRMS datasets limits effective metabolome coverage [29], while Du et al. [30], through the evaluation of 124 LC-HRMS metabolomics software tools, demonstrated that the lack of standardization among software platforms and databases remains a major obstacle to inter-study comparability.

Overall, these studies indicate that the computational layer currently represents one of the principal bottlenecks in translating metabolomics data into reliable biological knowledge.

### 2.1. Role of LC–MS Platforms in the Modular Pipeline

**Figure 2:**
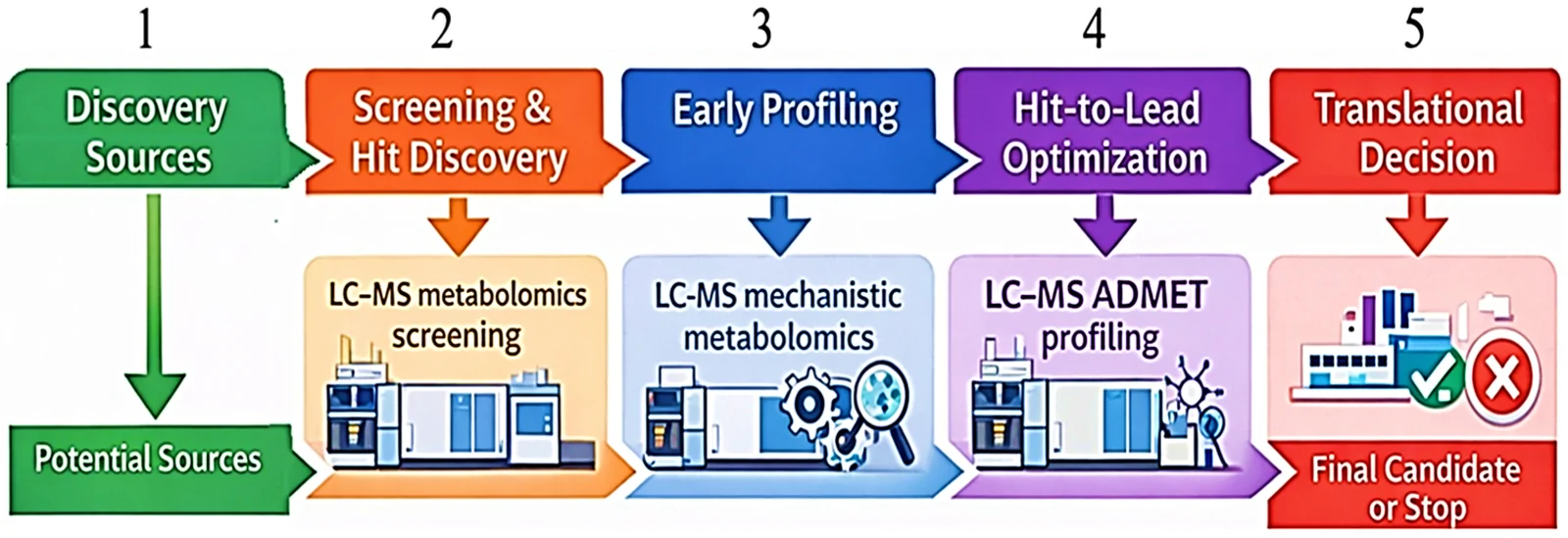
Role of LC–MS techniques across the stages of the antibacterial discovery pipeline: (1) discovery sources; (2) screening and hit discovery; (3) early profiling; (4) hit-to-lead optimization; and (5) translational decision-making.

LC-MS technologies perform distinct but interconnected functions throughout the different stages of the antibacterial discovery pipeline. Figure 2 illustrates how LC–MS platforms support all phases of the modern antibacterial discovery process in an integrated manner.

During the discovery-source phase (1) in Figure 2, untargeted metabolomics enables the identification of metabolites produced within complex systems such as multispecies biofilms. During screening and hit discovery (2) in Figure 2, metabolic profiling facilitates the identification of active compounds, early dereplication, and the generation of preliminary hypotheses regarding mechanisms of action. In the early profiling stage (3) in Figure 2, metabolic alterations are integrated with data on permeability, intracellular accumulation, and cytotoxicity, providing an initial functional characterization of the mechanism of action (MoA). During hit-to-lead optimization (4) in Figure 2, LC–MS platforms support the evaluation of pharmacokinetics, metabolic stability, and biotransformation of candidate compounds. Finally, in translational decision-making (5) in Figure 2, the integration of multi-omics and pharmacological data guides the selection of compounds with the highest probability of clinical success.

### 2.2. Complementary Techniques and Multi-Level Integration

High-resolution analytical technologies are essential for metabolite discovery and characterization because of their high sensitivity, accuracy, and broad analytical coverage. Owing to these characteristics, MS-based approaches are particularly valuable in untargeted metabolomics studies. Within this framework, several complementary technologies contribute to improving data interpretation and structural resolution.

IM-MS introduces collision cross section (CCS)-based separation, improving discrimination among isomeric species and increasing confidence in structural identification. The work of Paglia & Astarita [31] represents a key reference for understanding how CCS-based separation enhances the resolution of complex metabolites and lipids. However, the broader application of CCS-assisted annotation remains limited by the incomplete coverage of publicly available CCS databases, such as CCSbase, AllCCS, and MetCCS. Although these resources have substantially expanded CCS annotation, they still provide limited representation of microbial metabolites, particularly those originating from the oral microbiome. Consequently, many metabolites detected in oral biofilms lack reliable CCS reference values, reducing the effectiveness of library-based identification and highlighting the need for experimentally validated CCS libraries specifically tailored to oral microbial metabolites.

Imaging mass spectrometry (IMS) techniques such as MALDI and DESI enable the direct visualization of the spatial distribution of metabolites within biological tissues and biofilms [32]. In this context, MALDI-IMS is particularly suited for structured and minimally hydrated substrates such as mature dental plaque, biofilm layers and tooth surfaces, where matrix-assisted co-crystallization enhances spatial resolution. Conversely, DESI-IMS is more appropriate for highly hydrated or soft matrices, including saliva films and early-stage plaque deposits, enabling ambient ionization without extensive sample preparation.

This capability is particularly relevant in the oral environment, where the three-dimensional architecture of biofilms strongly influences metabolic interactions.

However, practical in situ IMS analysis of oral biofilms presents several challenges. The three-dimensional structure and dense extracellular polymeric matrix (EPS) of mature biofilms can reduce ionization efficiency, complicate matrix deposition in MALDI, and hinder uniform desorption in DESI. Sample preparation steps such as controlled sectioning, optimized matrix spraying and gentle surface stabilization are often required to preserve spatial integrity. Moreover, in situ imaging of native dental biofilms differs substantially from imaging of reconstructed in vitro biofilms. While in vitro models provide highly controlled experimental conditions that facilitate mechanistic studies and method optimization, in situ imaging preserves the native three-dimensional architecture, microbial diversity, and metabolic gradients of clinical biofilms, thereby providing a more realistic representation of the oral ecosystem. However, this increased biological relevance comes at the expense of greater technical complexity, as enamel hardness, hydration gradients, surface roughness, and the extracellular matrix can adversely affect ion yield and spatial resolution. Several optimization strategies—including tailored matrix formulations, cryo-sectioning and surface-compatible DESI solvents—have been developed to mitigate these limitations and improve metabolite detectability in oral biofilms.

In the context of highly heterogeneous ecosystems, in silico molecular annotation tools, including the software SIRIUS, which infers molecular formulas through fragmentation tree analysis [33]; CSI:FingerID, which predicts molecular structures using machine learning-derived fingerprints; and CANOPUS, which assigns compound classes via deep learning models, have proven valuable for structural prediction from MS/MS spectra thereby accelerating metabolite identification and prioritization [34]. Nevertheless, these approaches primarily generate predictive hypotheses that still require experimental validation, particularly when dealing with highly complex spectra.

Table 1 summarizes the main analytical techniques currently employed in the antibacterial discovery pipeline, highlighting their principal roles, strengths, and limitations.

Against this background, the oral microbiome represents an ideal model in which advanced analytical technologies can be integrated throughout the discovery pipeline, enabling the identification of novel antibacterial molecules, functional biomarkers, and metabolic vulnerabilities within complex multispecies biofilms.

## 3. The Oral Microbiome as a Source of Bioactive Molecules and Antimicrobial Resistance

The microbial community of the oral cavity is complex and highly organized. Microorganisms live in close proximity and establish synergistic and antagonistic interactions that determine health or disease [35]. Under balanced conditions, these interactions generate a stable and resilient network that maintains oral homeostasis and protects against environmental perturbations. When pressures such as a high-sugar diet or inflammation exceed a critical threshold, the balance is disrupted, leading to dysbiosis and favoring dental caries or periodontal disease [36]. Although the oral microbiome also comprises fungi, archaea, and viruses that participate in these ecological networks, the present review focuses primarily on bacterial communities. This choice reflects the current state of MS-based antibacterial discovery, which has been developed predominantly around bacterial metabolites, proteins, and biofilm-associated mechanisms. Accordingly, particular emphasis is placed on microbially derived molecules as potential sources of novel antibacterial agents, whereas host-derived molecules are discussed mainly as biomarkers and indicators of host–microbiome interactions that may guide antibacterial target discovery.

Interactions within the oral biofilm include adhesion and co-adhesion, nutritional cooperation, cell–cell signaling, horizontal gene transfer, and competition mediated by antimicrobial compounds [37]. Effective prevention therefore requires understanding microbial functions rather than taxonomic composition alone [38].

**Figure 3:**
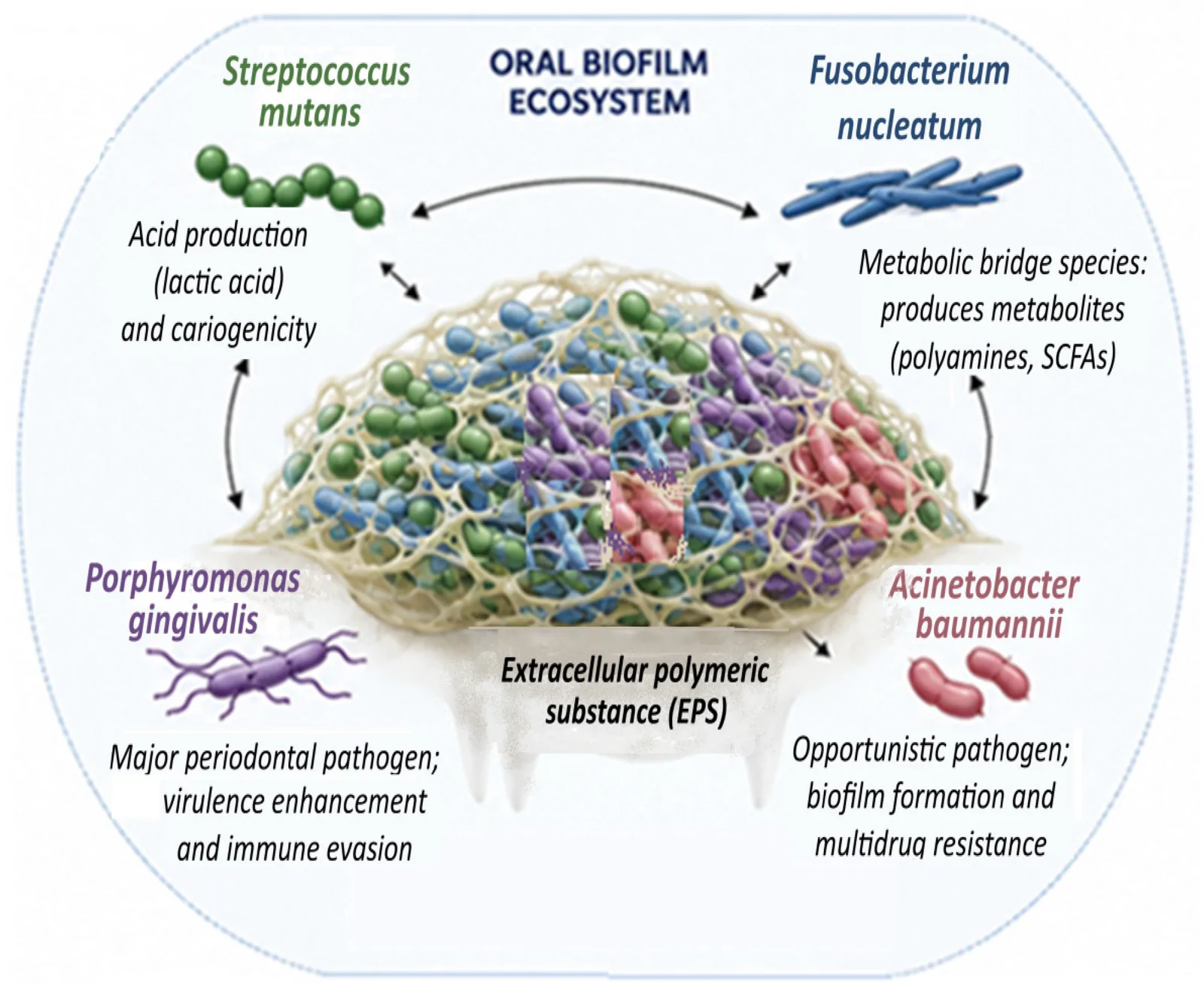
Schematic representation of the oral microbiome as a multispecies ecosystem.

The complexity of the oral cavity makes it a reservoir of antimicrobial resistance, but also a promising and still underexplored source of bioactive molecules for antibacterial discovery [17,39,40]. Figure 3 schematically illustrates the oral microbiome as a chemical ecosystem acting both as a source of bioactive metabolites and as a hub for antimicrobial resistance.

The mouth hosts very different microbial communities, and the study by Kim et al. [36] clarifies its spatial organization. Using advanced imaging techniques on teeth affected by caries in children, the researchers identified a microbial community organized into a three-dimensional, crown-shaped structure in which *Streptococcus mutans* is not only associated with caries but actively builds this architecture by producing an extracellular matrix that organizes the other microbes around it. This structure acts as a protective barrier against antimicrobials and creates acidic environments, promoting the progression of the disease. This study provided important insights into the functional organization of the oral microbiome.

The spatial structure of the microbiome modulates virulence and host–pathogen interaction in situ. More recently, Jiang et al. [41] further confirmed the role of *S. mutans* as a model organism exhibiting acidogenic and aciduric adaptation strategies, which modify local pH and select resistant communities. Additional metaproteomic evidence from oral–systemic disease studies [42] similarly underscore the functional relevance of *S. mutans* within complex microbial networks. Nevertheless, the specific mechanisms regulating its growth dynamics and virulence modulation remain to be clarified.

Other key oral pathogens include *Porphyromonas gingivalis* and *Fusobacterium nucleatum* which, in addition to playing a decisive role in the pathogenesis of periodontal diseases [43], have also been implicated in systemic diseases and tumor-associated processes [44,45,46]. For example, *F. nucleatum* models the metabolic microenvironment of the oral biofilm, producing metabolites that increase the virulence of *P. gingivalis*, which in this enhanced microenvironment alters the autophagy of oral epithelial cells, favoring the progression of oral squamous carcinoma (OSCC) [46]. However, although these associations are supported by numerous studies, their causal role remains a matter of debate and needs further functional validation.

In addition to well-established oral pathogens, opportunistic species such as *Acinetobacte baumannii* are increasingly recognized within the oral microbiome, particularly in dysbiotic conditions and vulnerable patient populations [47,48]. Although its presence in the oral cavity is less extensively characterized, its ability to form biofilms, persist in harsh environments and develop multidrug resistance suggests that it may act as a reservoir of antimicrobial resistance in oral niches. Emerging studies also indicate potential links with oral inflammatory processes and microenvironmental alterations [49], although these associations remain preliminary. At the molecular level, quorum-sensing systems such as abaR/abaL regulate virulence and biofilm formation, and recent metabolomics-based investigations have begun to explore the small-molecule repertoire associated with these pathways [50]. However, most available evidence derives from genomic and in vitro studies, underscoring the need for integrative approaches, particularly LC-MS-based workflows, that can connect resistance determinants with functional metabolic activity in complex biofilm environments.

Cooperative and competitive interactions among these key species contribute to the formation of metabolic micro-niches, the exchange of antimicrobial-resistance genes and the production of bioactive metabolites implicated in dysbiosis and disease progression. These features collectively highlight that antibacterial discovery in the oral cavity cannot rely on simplified monoculture systems but requires analytical strategies capable of capturing multispecies and dynamic metabolite-mediated interactions. LC-MS-based platforms enable the characterization of these interactions at the proteomic and metabolomic level, supporting the identification of biomarkers, metabolic vulnerabilities and new antibacterial candidates.

### 3.1. Analytical Techniques in the Modern Pipeline of Oral Antibacterial Discovery

The integration of advanced analytical techniques has profoundly transformed the modern pipeline of antibacterial discovery, allowing us to systematically explore the metabolic space of microbial communities and to understand the chemical interactions that characterize complex ecosystems such as oral biofilm. In this context, LC-MS-based untargeted metabolomics represents one of the most powerful tools for the identification of bioactive metabolites even in the absence of preliminary information on their structure or function [51,52]. This approach is particularly relevant in the study of the oral microbiome, where multispecies interactions generate considerable metabolic and functional diversity.

LC-MS/MS and LC-HRMS platforms can be applied across proteomic, metabolomic, and lipidomic workflows, offering an integrated view of the chemical micro-niches present within the oral biofilm.

Table 2 summarizes some relevant examples of theability of MS-based platforms to achieve a high analytical coverage of the oral proteome and metabolome, while allowing the detection of analytes present at very low abundance in different biological matrices of the oral cavity.

The detected molecular features comprise both host-derived and microbiome-derived molecules. Within the context of this review, particular emphasis is placed on microbially derived metabolites and proteins because of their potential as sources of novel antibacterial compounds and therapeutic targets, whereas host-derived molecules are primarily discussed as biomarkers and indicators of host–microbiome interactions.

Pioneering studies of high-depth salivary proteomics have demonstrated the ability of LC-MS/MS to characterize thousands of human and microbial proteins, establishing an important methodological reference for subsequent research [53]. Subsequently, integrated multi-omics approaches have shown how metabolic and protein signatures reflect the functional status of the oral microbiome and its interaction with the host [11,18,54,55]. This perspective is further supported by recent reviews highlighting the diagnostic potential of MS-based analyses in non-invasive biofluids [57] and the broader contribution of volatilomics to biomarker discovery in complex diseases [58].

Saliva emerges as a matrix of great diagnostic and biological interest, as it dynamically reflects both the metabolic activity of the microbiome and the physiological responses of the host.

Gardner et al. [59] provided a comprehensive methodological overview of the state of the art in salivary metabolomics, highlighting the central role of LC-MS and NMR in the identification of biomarkers associated with oral and systemic conditions. However, the authors also point to important methodological criticalities, including the heterogeneity of collection protocols, pre-analytical variability and the limited integration between microbiomics and metabolomics data.

Along these lines, Teruya et al. [60] applied non-targeted LC-MS approaches to the study of oral aging, identifying significant alterations in metabolites involved in glycolysis, amino acid metabolism, and the pentose-phosphate pathway. The results suggest a coordinated decline in oral cavity metabolic functions with age, although the limited sample size and geographical homogeneity reduce the generalizability of the conclusions.

Similarly, Grocholska et al. [61] provided a comprehensive methodological review on the application of mass spectrometry to saliva, critically describing workflows, sample preparation strategies, and major analytical limitations.

More recently, Ciurli et al. [62] introduced an innovative spatiotemporal mapping approach of the oral metabolome, demonstrating how different areas of the oral cavity present distinct and dynamic metabolic profiles. This type of approach goes beyond the concept of “average saliva” and allows a more realistic representation of the ecological heterogeneity of the oral cavity. However, the complexity of sampling and the high biological variability still make it difficult to standardize and clinically translate these methodologies.

The functional importance of LC–MS analyses emerges clearly in studies dedicated to metabolic interactions within multispecies biofilms. Sakanaka et al. [63] demonstrated how *F. nucleatum* acts as a central metabolic node, converting substrates derived from commensal species into bioactive metabolites capable of modulating the virulence of *P. gingivalis*. These results support the concept of metabolic cross-feeding as a fundamental determinant of biofilm resilience and its ability to adapt to antimicrobial stresses. In parallel, metaproteomic studies have shown that altered proteolytic and metabolic activities are associated with dysbiotic states, such as dental erosion and periodontal disease progression [54].

Another major contribution of LC–MS technologies is the exploration of the so-called “cryptic chemical space” of the oral microbiome. Edlund et al. [13] showed that numerous bioactive metabolites are produced exclusively in multispecies settings and are absent in traditional monoclonal cultures. This paradigm has significantly expanded the landscape of antibacterial discovery, highlighting the potential of the oral microbiome as a source of novel bioactive molecules. However, the structural identification, isolation and functional validation of these metabolites remain complex processes and still represent one of the main bottlenecks in the discovery pipeline.

In parallel, LC-MS-based proteomics has gained increasing relevance in the identification of salivary biomarkers associated with oral and systemic diseases. Recent systematic reviews [55,56,64] show how these platforms allow the identification of differentially expressed proteins and correlate them with specific disease states. However, important challenges remain, including the heterogeneity of sampling protocols, small sample sizes, the absence of independent validation cohorts, and the variability of bioinformatics pipelines. Even methodological frameworks such as QUADAOMICS, which integrate metagenomic, metatranscriptomic, metaproteomic, and metabolomic layers to improve the quality assessment of omics studies, do not eliminate the risk of bias and overinterpretation of results.

The most recent evolution in the field concerns the integration between LC-MS and IMS techniques, which introduce a spatial dimension into metabolomic analysis. Unlike traditional extraction approaches, these techniques allow direct visualization of the distribution of metabolites within the three-dimensional matrix of the biofilm, preserving the ecological context of microbial interactions. Although the sensitivity and quantification are still lower than conventional LC-MS, IMS allows the identification of metabolites located in specific micro-niches characterized by nutrient gradients, oxygen and environmental stresses.

Separately, IM–MS-based approaches add an additional layer of structural separation through CCS measurement, improving the distinction between isomeric species and supporting molecular identification [65]. The principles described by Paglia & Astarita [31] have been applied, for example, in the study by Vaysse et al. [66], in which lipid profiles obtained by Rapid Evaporative Ionization Mass Spectrometry (REIMS) were used to distinguish tumor and healthy tissues of the oral cavity.

The integration between spatial data derived from IMS and multi-omics data obtained by LC-MS is progressively redefining the study of oral biofilms under clinically realistic conditions. Studies such as those of Bostanci et al. [11] and Overmyer et al. [67] show that such approaches allow the identification of molecular signatures associated with diabetes, oral inflammation and periodontitis, highlighting a close relationship between spatial organization of the biofilm and disease status. In addition, these methodologies allow us to monitor the distribution of antibiotics and antimicrobial agents within the extracellular matrix, highlighting phenomena of limited penetration and localized resistance that do not emerge in planktonic models.

Overall, LC-MS-based technologies are fundamental tools for understanding the chemical and ecological complexity of the oral cavity. The integration between metabolomics, proteomics, lipidomic and spatial imaging now enables the direct integration of metabolic production, biofilm organization, and microbial adaptation, opening new perspectives for the identification of biomarkers, metabolic vulnerabilities and innovative antibacterial compounds. Despite significant progress, crucial challenges remain related to the standardization of workflows, the annotation of unknown metabolites and the biological validation of results, aspects that will be decisive in translating these technologies into truly applicable tools in antibacterial discovery and precision oral medicine. In addition, effective multi-omics integration depends on robust data-processing strategies, including batch-effect correction, signal-drift adjustment, normalization and feature-alignment procedures. The increasing use of machine-learning and AI-based approaches for dimensionality reduction, feature selection and predictive modeling further supports the integration of metabolomic and proteomic datasets into coherent biological interpretations.

### 3.2. Representative Oral Microbiome-Derived Molecules Identified Through MS-Based Approaches

Representative examples of bioactive metabolites and biomarkers identified through MS-based approaches are summarized in Table 3.

The analytical platforms highlighted metabolic vulnerabilities, ecological signals of various classes of bioactive molecules that may be relevant for future antibacterial discovery. Table 4 summarizes the biological relevance and analytical techniques relevant to the discovery.

Recent MS-based studies indicate that the oral microbiome is not only a reservoir of antimicrobial resistance, but also a source of bioactive metabolites with therapeutic activity. Multispecies biofilms have been shown to produce cryptic metabolites and metabolic interaction networks that remain undetectable in monoculture systems [13,63]. These findings highlight the translational potential of oral microbiome-derived molecules as candidate antibacterial or anti-biofilm agents. Representative examples identified through MS-based approaches are summarized in Table 5.

Overall, these findings highlight the translational potential of oral microbiome-derived metabolites and support the integration of MS-based multi-omics approaches into future antibacterial discovery pipelines.

### 3.3. Functional Prioritization of Antibacterial Metabolites

Beyond the identification of novel metabolites, an equally important step in the antibacterial discovery pipeline is the functional characterization of their biological activity. Structural annotation alone is insufficient to prioritize compounds for drug development, making the elucidation of their mechanisms of action (MoA) a critical component of the discovery process. In the oral microbiome, MS-based metabolomics has revealed metabolites that interfere with bacterial physiology through multiple mechanisms, including disruption of biofilm formation, modulation of quorum sensing, metabolic competition, inhibition of virulence-associated pathways, and direct antibacterial activity. From a biochemical perspective, these metabolites exert their antibacterial effects through different mechanisms of action. Some directly inhibit bacterial growth by disrupting membrane integrity, impairing cell-wall biosynthesis, or interfering with protein and nucleic-acid synthesis. Others primarily act through anti-virulence mechanisms, including inhibition of quorum sensing, suppression of extracellular polymeric substance (EPS) production, disruption of biofilm maturation, interference with metabolic pathways required for pathogen persistence, or sequestration of essential nutrients such as iron. Increasing evidence suggests that many orally derived metabolites attenuate bacterial fitness and ecological competitiveness rather than causing direct cell death, thereby reducing selective pressure for antimicrobial resistance while preserving the overall balance of the resident microbiota. This ecological mode of action is particularly attractive for oral antibacterial discovery because it targets the functional organization of dysbiotic biofilms rather than indiscriminately eliminating microbial populations. The metabolic cross-feeding described by Sakanaka et al. [63] illustrates how metabolites such as polyamines and short-chain fatty acids regulate the physiology and virulence of *P. gingivalis* through interspecies metabolic interactions rather than direct killing. Similarly, the work of Cleaver et al. [54] identified altered proteolytic activities associated with oral dysbiosis, highlighting enzymatic pathways that may represent novel antibacterial targets. These examples demonstrate that MS-based discovery increasingly identifies ecological vulnerabilities within multispecies biofilms, expanding the concept of antibacterial activity beyond growth inhibition alone. Consequently, integrating metabolite identification with functional assays, mechanism-of-action studies and biofilm validation has become an essential step for prioritizing antibacterial leads, reducing false-positive candidates and improving the translational potential of MS-guided discovery pipelines.

## 4. Future Integrated Pipeline for Oral Antibacterial Discovery

**Figure 4:**
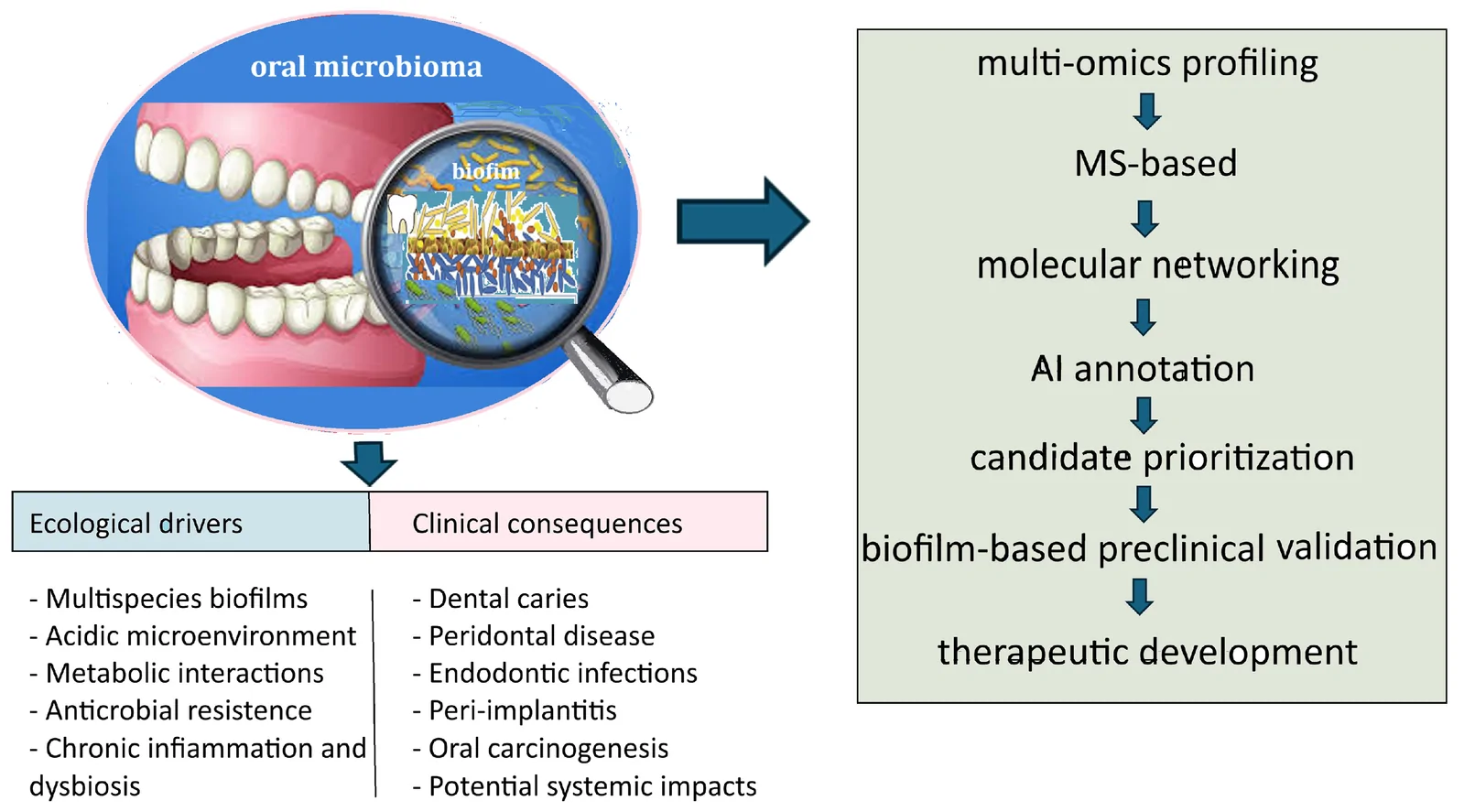
Proposed evolution of the oral antibacterial discovery pipeline integrating advanced MS-based multi-omics technologies, computational analysis, and translational validation to link ecological drivers with clinical consequences. The downstream stages, including biofilm-based preclinical validation and therapeutic development, represent iterative and multidisciplinary workflows encompassing lead optimization, mechanistic studies, multispecies biofilm testing, and translational assessment.

The convergence of spatial metabolomics, multi-omics integration, advanced mass spectrometry, and computational analysis is progressively reshaping antibacterial discovery in the oral cavity. Building on the evidence discussed throughout this review, Figure 4 illustrates a future integrated antibacterial discovery pipeline, highlighting the interplay between microbial ecology, advanced analytical platforms, bioinformatics, and translational applications.

Collectively, the studies reviewed indicate a progressive transition from reductionist antibacterial discovery strategies toward integrated, ecology-driven workflows that better reflect the biological complexity of the oral microbiome. By combining analytical depth, spatial resolution, functional multi-omics, and computational prediction, these approaches have the potential to improve target prioritization, accelerate the identification of bioactive molecules, and facilitate the translation of laboratory discoveries into clinically relevant therapeutic strategies for oral infectious diseases.

## 5. Conclusions

The discovery of new antibacterial agents remains one of the most pressing priorities in the global response to antimicrobial resistance. The evidence reviewed here shows that the oral cavity is far more than a site of infection: it represents a highly dynamic microbial ecosystem in which complex ecological interactions, specialized metabolism, and antimicrobial resistance coexist. These characteristics make the oral microbiome a unique environment for exploring novel antibacterial targets and previously unrecognized bioactive molecules.

Recent advances in mass spectrometry, metabolomics, proteomics, imaging technologies, and multi-omics integration have profoundly expanded our ability to investigate this complexity. By linking microbial composition with metabolic function, spatial organization, and biofilm activity, these analytical approaches provide a systems-level understanding of oral microbial ecology that cannot be achieved using conventional culture-based strategies alone. Nevertheless, important challenges remain, including workflow standardization, metabolite annotation, functional validation, and the translation of analytical discoveries into clinically relevant antibacterial candidates. Importantly, the identification of a metabolite does not necessarily imply biological relevance or therapeutic potential. Bridging the gap between molecular annotation and biological validation remains one of the principal bottlenecks in MS-guided antibacterial discovery. Moreover, many current multi-omics studies remain predominantly associative, highlighting the need for functional studies to establish causal relationships between microbial metabolites, ecological interactions, and antibacterial activity.

To provide a clearer synthesis of the three major application directions discussed in this review, the main conclusions can be summarized as follows:

(i) Antibacterial molecule discovery.

Advanced MS-based metabolomics and proteomics enable the identification of chemically diverse bioactive metabolites, metabolic vulnerabilities and ecological interactions that can be exploited for next-generation antibacterial agents. The oral microbiome emerges as a reservoir of novel molecular scaffolds and adaptive pathways relevant to antibacterial innovation. These findings support the growing interest in ecological modulation of dysbiotic biofilms as a complementary strategy to conventional broad-spectrum antimicrobial therapy.

(ii) Monitoring antimicrobial resistance.

Proteomic and metabolomic signatures support the detection of resistance determinants, ARG transfer and biofilm-associated phenotypes. MS-based workflows offer sensitive tools for tracking the emergence, dissemination and functional expression of antimicrobial resistance within complex oral communities.

(iii) Disease diagnosis and clinical stratification.

High-resolution analytical platforms facilitate the characterization of diagnostic biomarkers linked to dysbiosis, inflammation and biofilm activity. Integrating spatial metabolomics, imaging MS and multi-omics data enhances the ability to distinguish disease states and identify clinically meaningful molecular patterns.

Overall, the literature reviewed indicates that the evolution of advanced analytical methodologies is progressively transforming antibacterial discovery into an integrated, ecology-driven process. Rather than being viewed solely as a reservoir of antimicrobial resistance, the oral microbiome should also be considered a valuable source of chemically diverse and biologically active molecules. Systematic exploration of this ecosystem using advanced MS-based analytical platforms, realistic biofilm models, and computational approaches may accelerate the discovery of next-generation antimicrobial agents, not only for the treatment of oral infectious diseases but also as a broader source of innovative therapeutics to combat antimicrobial resistance.

This review has several limitations. First, the coverage of the literature is necessarily selective and reflects the availability of studies employing MS-based approaches in oral microbiome research; emerging technologies such as single-cell mass spectrometry, native MS or ultrahigh-throughput metabolomics were not included because their application to oral ecosystems remains limited. Second, most of the evidence discussed derives from in vitro or early-stage investigations, and the translation of these findings to clinical settings is still in progress. In addition, considerable methodological heterogeneity across studies, including differences in sampling strategies, biofilm models, extraction protocols, and bioinformatic workflows, limits reproducibility and hampers direct comparison of results across investigations. Finally, the rapid evolution of analytical platforms and computational tools means that some methodological advances may not yet be fully represented. These limitations highlight the need for continued methodological development and broader integration of next-generation MS technologies in future studies. Future progress will therefore depend not only on continued technological innovation, but also on the development of standardized analytical workflows, comprehensive microbial spectral databases, experimentally validated biofilm models and interdisciplinary strategies capable of translating molecular discoveries into clinically useful antibacterial therapies.

## Figures and Tables

**Table 1:** Analytical techniques employed in the antibacterial discovery pipeline: principal roles, strengths, and limitations.

Technique	Main Role	Strengths	Limitations
LC-MS/MS (HRMS, Q-TOF)	Metabolite discovery and characterization	High sensitivity, sub-ppm accuracy, broad analytical coverage	Ionization dependency, data complexity
LC-MS	Exploration of chemical space	Identification of unknown metabolites	Variability, incomplete databases
IM-MS	Structural separation (CCS-based)	Isomer/isobar discrimination	Incomplete CCS reference databases for microbial metabolites; analytical complexity
IMS (MALDI, DESI)	Spatial metabolite distribution	In situ analysis	Lower sensitivity, limited metabolite identification
**In silico annotation (SIRIUS, CSI:FingerID, CANOPUS)**	Structural prediction	Speed, prioritization support	False positives, need for experimental validation

**Table 2:** Comparative overview of oral matrices analyzed by MS-based approaches, highlighting analytical strengths, limitations and their relevance for antibacterial discovery.

Matrix	Main Analytes Detected	Molecular Origin	Analytical Depth/Sensitivity	Relevance for Antibacterial Discovery	Critical Considerations	Relevant Ref.
Saliva	Human proteins, microbial proteins, posttranslational peptides	Mixed (host + microbial)	Ultra-deep depth proteomics (>3000 proteins); high sensitivity for low abundance proteins	Biomarker discovery and prioritization of host–microbiome interactions relevant to antibacterial target selection	High biological variability, dilution effects and difficulty in discriminating host- from microbially derived molecules	[53]
Saliva + dental plaque	Human and microbial proteins; metabolites; lipids	Mixed (host + microbial; microbial enrichment in plaque)	Simultaneous multi-omics profiling; high sensitivity	Identification of microbial pathways associated with dysbiosis and potential therapeutic vulnerabilities	Sampling and extraction protocols differ substantially among studies, limiting comparability	[11,18]
Saliva, biofilm, eroded tooth surfaces	Proteases, bacterial proteins, metabolites	Predominantly microbial (biofilm)	Deep metaproteomics + metabolomics. Excellent sensitivity to rare microbial enzymes and underrepresented pathways	Identification of disease-associated metabolic activities and potential antibacterial vulnerabilities	Mostly observational; most findings remain associative and require functional validation	[54]
Saliva/plaque	Proteins, metabolites, lipids	Mixed	Deep multi- omics characterization	Identification of disease-associated protein and metabolic signatures supporting target prioritization and biomarker discovery	Correlations do not necessarily demonstrate causal mechanisms	[55]
Oral biofilm, microbial cultures	Bacterial metabolites, semi-polar compounds, unknown molecules	Microbial	Detection of thousands of LC–MS features, including cryptic metabolites	Primary source of novel antibacterial metabolites; enables discovery of ecological interactions	Large proportion of detected metabolites remain unannotated; structural and functional validation is challenging	[13]
Saliva/Biofilm	Metabolites, proteins, lipids	Mixed	High metabolic coverage. Detection of bacterial metabolites and chemical fingerprints	Characterization of metabolic fingerprints associated with oral physiological and pathological states.	Biological significance of many low-abundance metabolites remains uncertain	[56]

**Table 3:** Major classes of bioactive metabolites and biomarkers identified in the oral microbiome.

Class	Representative Example	Biological Role
Bioactive metabolites	Short-chain fatty acids	Inflammation modulation
	Polyamines	Biofilm resilience
	Small peptidic molecules	Antibacterial potential
	Siderophore-like metabolites	Competition and virulence
Proteomic biomarkers	Inflammatory salivary proteins	Periodontitis
	Proteolytic enzymes	Dysbiosis
	Oxidative stress metabolites	Caries/OSCC
Space metabolites	Localized lipid signatures	Spatial metabolic heterogeneity
	REIMS-derived lipid fingerprints	Oral cancer discrimination

**Table 4:** MS-based analytical workflows supporting oral antibacterial discovery.

Bioactive Molecule/Biomarker	Oral Source/Species	Biological Significance	Analytical Platform	Potential Application	Reference
Small peptidic metabolites	Multispecies oral biofilm	Previously uncharacterized secreted metabolites involved in microbial interactions	LC–MS/MS + molecular networking	Discovery of novel bioactive scaffolds	[13]
Metabolic cross-feeding metabolites	*F. nucleatum*/*P. gingivalis*	Virulence modulation and biofilm resilience	LC–MS metabolomics	Identification of metabolic vulnerabilities in oral biofilms	[63]
Salivary inflammatory and microbial proteins	Periodontitis-associated saliva and plaque	Differential protein expression associated with periodontal inflammation	LC–MS/MS proteomics	Salivary biomarker discovery	[11,56,64]
Proteolytic enzymes and dysbiosis-associated metabolites	Erosive oral biofilms	Altered metabolic and proteolytic activity associated with oral dysbiosis	Metaproteomics + metabolomics	Functional biomarkers of biofilm-associated disease	[56]
Lipidomic fingerprints	Oral cancer tissues	Tumor discrimination	REIMS/IM–MS	Surgical guidance/diagnostics	[66]
Antimicrobial resistance genes (ARGs) and mobile genetic elements	Oral biofilm communities	Reservoir and dissemination of antimicrobial resistance	Metagenomics + LC–MS-integrated approaches	Resistance surveillance and target prioritization	[14,15]
Spatial metabolomic profiles	Distinct oral cavity niches	Site-specific metabolic heterogeneity	LC–MS spatial metabolomics	Precision oral diagnostics and ecological profiling	[62]

**Table 5:** Representative oral microbiome-derived molecules with therapeutic activity identified through MS-based approaches.

Molecule/ Family	Producing Species	Functional Role	Potential Relevance	Reference
Mutanobactins	*S. mutans*	Oxidative stress adaptation and biofilm fitness	Anti-biofilm targeting	[68,69]
Reuterin-like metabolites	Oral *Lactobacillus* spp.	Antimicrobial activity	Ecological therapeutics	[70]
Hydrogen peroxide	Oral *Streptococcus* spp.	Competitive inhibition	Colonization resistance	[71]
Autoinducer-2 (AI-2)	Multispecies biofilms	Quorum sensing	Targets for biofilm disruption	[72]
Polyamines	Dysbiotic biofilms	Stress adaptation	Metabolic targeting	[62,73]
SCFAs	Periodontal anaerobes	Inflammation modulation	Host–microbiome modulation strategies	[11,74]

## Data Availability

No new data were created or analyzed in this study. Data sharing is not applicable to this article.
